# Transcriptome Profiling of Bovine Milk Oligosaccharide Metabolism Genes Using RNA-Sequencing

**DOI:** 10.1371/journal.pone.0018895

**Published:** 2011-04-25

**Authors:** Saumya Wickramasinghe, Serenus Hua, Gonzalo Rincon, Alma Islas-Trejo, J. Bruce German, Carlito B. Lebrilla, Juan F. Medrano

**Affiliations:** 1 Department of Animal Science, University of California Davis, Davis, California, United States of America; 2 Department of Chemistry, University of California Davis, Davis, California, United States of America; 3 Department of Food Science and Technology, University of California Davis, Davis, California, United States of America; Wellcome Trust Centre for Stem Cell Research, United Kingdom

## Abstract

This study examines the genes coding for enzymes involved in bovine milk oligosaccharide metabolism by comparing the oligosaccharide profiles with the expressions of glycosylation-related genes. Fresh milk samples (n = 32) were collected from four Holstein and Jersey cows at days 1, 15, 90 and 250 of lactation and free milk oligosaccharide profiles were analyzed. RNA was extracted from milk somatic cells at days 15 and 250 of lactation (n = 12) and gene expression analysis was conducted by RNA-Sequencing. A list was created of 121 glycosylation-related genes involved in oligosaccharide metabolism pathways in bovine by analyzing the oligosaccharide profiles and performing an extensive literature search. No significant differences were observed in either oligosaccharide profiles or expressions of glycosylation-related genes between Holstein and Jersey cows. The highest concentrations of free oligosaccharides were observed in the colostrum samples and a sharp decrease was observed in the concentration of free oligosaccharides on day 15, followed by progressive decrease on days 90 and 250. Ninety-two glycosylation-related genes were expressed in milk somatic cells. Most of these genes exhibited higher expression in day 250 samples indicating increases in net glycosylation-related metabolism in spite of decreases in free milk oligosaccharides in late lactation milk. Even though fucosylated free oligosaccharides were not identified, gene expression indicated the likely presence of fucosylated oligosaccharides in bovine milk. Fucosidase genes were expressed in milk and a possible explanation for not detecting fucosylated free oligosaccharides is the degradation of large fucosylated free oligosaccharides by the fucosidases. Detailed characterization of enzymes encoded by the 92 glycosylation-related genes identified in this study will provide the basic knowledge for metabolic network analysis of oligosaccharides in mammalian milk. These candidate genes will guide the design of a targeted breeding strategy to optimize the content of beneficial oligosaccharides in bovine milk.

## Introduction

Oligosaccharides are present in bovine milk as lactose-derived free forms and as glycoconjugates bound to proteins and lipids [1]. Recent studies on free milk oligosaccharides and glycoconjugates demonstrate both local and systemic beneficial effects to the suckling human neonate [2], [3], [4]. Milk oligosaccharides can withstand the pH of the stomach and resist enzymatic digestion in the gastrointestinal tract [5]. Intact oligosaccharides serve as a prebiotic to beneficial bacterial populations in the intestines, especially the *Bifidobacterium bifidum* genus localized in the colon [6]. Milk oligosaccharides and glycoproteins have demonstrated protective qualities against enteric pathogen infections in infants by enhancing the binding of IgA with pathogens, having an antibacterial activity against pathogens and competing with pathogen binding sites [7], [8], [9]. There is considerable evidence that breastfeeding provides long-term cognitive advantages to the infant, even through adulthood [10]. It has been shown in developing piglets that sialic acid supplementation of a milk formula acts as a conditional nutrient during periods of rapid brain growth [11]. Therefore, it is thought that large amounts of sialylated oligosaccharides in human milk are responsible for the higher cognitive performance of breast-fed infants [11].

Quantification of total oligosaccharides in milk is difficult to achieve due to the structural complexity, variety of glycan structures, and multiple glycosylation sites in proteins and lipids [1]. However studies have been conducted on the composition of free milk oligosaccharides in several mammalian species. One liter of mature human milk contains approximately 5–10 g of lactose-derived free oligosaccharides [12], [13]. The concentration of free oligosaccharides in bovine milk is reported to be approximately 20-fold lower than in human milk oligosaccharides (HMO) [14], [15].

Due to the beneficial effects of milk oligosaccharides, it is important to develop the means to enhance the concentrations of beneficial oligosaccharides in cow milk. At present it is not possible to produce these compounds in the quantities and purities necessary for such applications as infant products using any other technology. Any strategy to improve the yield and composition of cow milk oligosaccharides must be based on a thorough understanding of oligosaccharide metabolism in the mammary gland and in milk itself. Even though the study of glycobiology is emerging as a revolution in biomolecular and biomedical fields [16], there is limited knowledge on the metabolism of oligosaccharides in mammalian milk. Toward the first step to understanding the complex biology of milk oligosaccharide metabolism, it is important to identify the genes that encode for glycosylation-related enzymes including glycosyltransferases, glycosidases and sugar transporters. Expression studies have been conducted on glycosylation-related genes in cell cultures and human tissue cells [17], [18] yet no studies have been conducted on expression profiling of glycosylation-related genes in milk.

Most gene expression studies on the bovine mammary gland have been developed using biopsy samples [19], [20]. A non-invasive sampling procedure was introduced by Boutinaud *et al.* [19] in which mRNA is isolated from somatic cells released into milk during lactation. Cánovas *et al.* [21] showed that extensive similarities exist between the mammary gland and milk somatic cell transcriptomes. Due to the non-invasive nature of milk somatic cell sampling, this protocol was used here to sample cows at different stages of lactation. In the present study the key genes of bovine milk oligosaccharide metabolism were identified by comparing the bovine milk oligosaccharide profile analysis using high-performance mass spectrometry, with the expression of glycosylation-related genes in milk measured by RNA-Sequencing.

## Materials and Methods

### Animals and sample collection

Four healthy Holstein cows and Jersey cows in their 2nd/3rd lactation at the UC Davis dairy were selected for the study. The animals were kept in free stall housing, fed with total mixed ration (TMR), and had access to water *ad libitum*. Cows were milked twice daily at 4 a.m. and 4 p.m. in the milking parlor and managed according to AAALAC (American Association for Accreditation of Laboratory Animal Care) guidelines. Sample collection was approved by the UC Davis Institutional Animal Care and Use Committee (IACUC), protocol #16151.

Thirty-two milk samples were collected by hand-milking the four quarters of eight cows (four animals per breed) at days 1 (colostrum), 15 (transition milk), 90 (mature milk) and 250 (late lactation milk) of lactation 3 hours after the morning milking. Milk samples were divided into two portions for oligosaccharide profile analysis and RNA extraction from somatic cells. Samples for oligosaccharide profile analysis were frozen at −20°C in 1.5 ml tubes.

### Oligosaccharide extraction from raw milk and deanomerization

Milk samples were completely thawed, and 0.5 ml was centrifuged at 4,000 *g* in a microfuge for 30 min at 4°C. After the top fat layer was removed, 4 volumes of chloroform/methanol (2∶1, vol/vol) and 2 volumes of deionized H_2_O were added to the defatted milk samples. After centrifugation at 4,000 *g* for 30 min at 4°C, the upper layer was carefully transferred to another tube. Two volumes of ethanol were added to the mixture and left overnight at 4°C, followed by a 30 min centrifugation at 4,000 *g* at 4°C to remove denatured protein. The supernatant (milk OS-enriched fraction) was freeze-dried using a speed vacuum. Reconstituted oligosaccharides were passed through a C8 cartridge in order to remove remaining protein.

In order to avoid chromatographic peak splitting due to anomeric forms, native milk OS were reduced to alditol forms. Oligosaccharide fractions were combined with aqueous sodium borohydride solution (final concentration 1.0 M) and incubated at 65°C for 1 hour.

### Oligosaccharide enrichment with graphitized carbon SPE

Oligosaccharides were purified by graphitized carbon (GC) solid-phase extraction. GC cartridges were washed with 0.10% (v/v) trifluoroacetic acid in 80% acetonitrile/water (v/v) followed by conditioning with water. The oligosaccharide fractions were loaded onto each cartridge and washed with water at a flow rate of 1 ml/min to remove salts and small mono-/disaccharides. Oligosaccharides were eluted with 20% acetonitrile/water (v/v) and dried *in vacuo* prior to MS analysis.

### Chromatographic separation of milk oligosaccharides

Milk oligosaccharides were reconstituted in water and analyzed using an Agilent HPLC-Chip Time-of Flight (Chip-TOF) Mass Spectrometry (MS) system equipped with a microwell-plate autosampler (maintained at 6°C), capillary sample loading pump, nano pump, HPLC-Chip/MS interface, and the Agilent 6210 TOF MS detector. The chip consisted of a 9×0.075 mm i.d. enrichment column and a 43×0.075 mm i.d. analytical column, both packed with 5 µm porous graphitized carbon (PGC) as the stationary phase. For sample loading, the capillary pump delivered 0.1% formic acid in 3.0% acetonitrile/water (v/v) isocratically at 4.0 µl/min. Injection volume was 2.0 µl for each sample. A nano pump gradient was delivered at 0.4 µl/min using (A) 0.1% formic acid in 3.0% acetonitrile/water (v/v) and (B) 0.1% formic acid in 90% acetonitrile/water (v/v). Samples were eluted with 0% B, 0.00–2.50 min; 0 to 16% B, 2.50–20.00 min; 16 to 44% B, 20.00–30.00 min; 44 to 100% B, 30.00–35.00 min; and 100% B, 35.00–45.00 min. This was followed by re-equilibration at 0% B. The drying gas temperature was set at 325°C with a flow rate of 4 L/min (2 L of filtered nitrogen gas and 2 L of filtered dry grade compressed air). MS spectra were acquired in the positive ionization mode over a mass range of *m*/*z* 400–2500 with an acquisition time of 1.5 seconds per spectrum. Mass correction was enabled using reference masses of *m*/*z* 622.029, 922.010, 1221.991 and 1521.971 (ESI-TOF Tuning Mix G1969-85000, Agilent Technologies, Santa Clara, CA).

### RNA extraction for gene expression studies

Milk cells were pelleted by adding 50 µl of 0.5 M EDTA to 50 ml of fresh milk and centrifuged at 1800 rpm at 4°C for 10 minutes. The pellet of cells was washed with 10 ml of PBS at pH7.2 and 10 µl of 0.5 M EDTA (final conc. 0.5 mM) and filtered though a sterile cheese cloth to remove any debris. The milk cells were then centrifuged again at 1800 rpm, 4°C for 10 minutes. The supernant was decanted and RNA was extracted from the milk cell pellet by the Trizol method (Invitrogen, Carlsbad, CA) according to the manufacturer instructions. RNA was quantified by ND-1000 spectrophotometer (Fisher Thermo, Wilmington, MA) and the quality and integrity was assessed by the spectrophotometer 260/280 ratio, gel electrophoresis and by capillary electrophoresis with an Experion bio-analyzer (Bio-Rad, Hercules, CA).

### RNA sequencing and data analysis

Gene expression analysis was conducted on Holstein and Jersey cows for day 15 (n = 3 per breed) and day 250 (n = 3 per breed) of lactation by Illumina RNA sequencing (RNA-Seq) technology. Messenger RNA was isolated and purified using RNA-Seq sample preparation Kit (Illumina, San Diego, CA). Then mRNA was fragmented to approximately 200 bp fragments and first and second strand cDNA were synthesized, followed by end repair and adapter ligation. The fragments were purified and sequenced at the UC Davis Genome Center DNA Technologies Core Facility using the Illumina Genome Analyzer (GAII). Short sequence reads of 36–40 bp were assembled, and analyzed in the RNA-Seq and expression analysis application of CLC Genomics workbench 3.7. (CLC Bio, Aarhus, Denmark). Bovine genome, Btau 4.0 (http://www.ncbi.nlm.nih.gov/genome/guide/cow/index.html) was utilized as the reference genome for the assembly. Data was normalized by calculating the ‘reads per kilo base per million mapped reads’ (RPKM) for each gene [22] and annotated with NCBI bovine genome assembly (27,368 unique genes). For the statistical analysis the samples were assumed to be independent of each other because of the 235 day interval between sample collections. A t-test was performed on log_2_-transformed data to identify the genes with significant changes in expression (p≤0.05 and FDR q≤0.3) between the two stages of lactation and between the two breeds.

In order to improve the annotation of glycosylation-related genes a *de novo* assembly and Gene Ontology (GO) annotation were performed using 33 million unmapped reads from Holstein cows. Contig sequences were generated using the CLC Genomics workbench 3.7 *de novo* assembly application. Consensus sequences were imported to Blast2GO program to perform the blastx, mapping and GO annotation [23]. Results were manually curated to identify novel glycosylation-related transcripts in bovine milk.

## Results and Discussion

### Analysis of the milk oligosaccharide profiles

**Figure 1:**
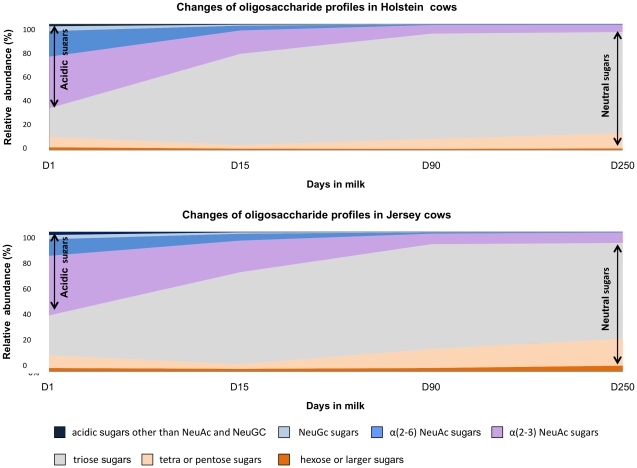
Changes in milk oligosaccharide profiles with the stages of lactation. Stacked area charts illustrating the changes in the types of free oligosaccharides present in Holstein (n = 4) and Jersey (n = 4) milk at different stages of lactation (D1: colostrum, D15: transition milk, D90: mature milk, D250: late lactation milk). The oligosaccharide levels are shown in percent relative abundance. With the progress of lactation, the anionic acidic oligosaccharides decreased in parallel to an increase in neutral oligosaccharides.

The building blocks of Holstein and Jersey milk oligosaccharides are glucose **(Glc)**, **galactose (Gal)**, *N*-acetylglucosamine **(GlcNAc)**, *N*-acetylgalactosamine **(GalNAc)**, N-acetylneuraminic acid **(NeuAc)** and *N*-glycolylneuraminic acid **(NeuGc)**. Remarkable similarity was observed between the milk oligosaccharide profiles of Holstein and Jersey cows at the same stage of lactation. The highest concentrations of free oligosaccharides were observed in the colostrum samples. A sharp decrease was observed in the concentration of free oligosaccharides in the transition milk, followed by a progressive decrease in the free oligosaccharides in mature and late lactation milk. The majority of the free oligosaccharides in the colostrum samples were anionic oligosaccharides containing NeuAc and NeuGc. However, with the progress of lactation, the anionic acidic oligosaccharides decreased in parallel to an increase in neutral oligosaccharides constituted mainly of galactosylated oligosaccharides (Figure 1). The results obtained from the present study were in agreement with the study conducted by Tao *et al.* [15] on Holstein and Jersey milk samples collected within 12 hrs of calving, day 6 and day 15 of lactation.

In colostrum, most of the free oligosaccharides were sialylated with 66.8% abundance in Holstein and 59.9% abundance in Jersey. The degree of sialylation decreased progressively with the stage of lactation reaching just 6.2% sialylation in late lactation Holstein milk and 7.9% sialylation in late lactation Jersey milk. Similar to humans, NeuAc was the main contributing sialic acid **(sia)** in bovine milk oligosaccharides. However, in contrast to humans, NeuGc-containing oligosaccharides were also identified in both Holstein and Jersey milk samples. In humans, NeuGc is not produced in any of the body tissues due to a frame-shift mutation early in human evolution leading to the deactivation of the CMP-NeuAc-hydroxylase **(** ***CMAH*** **)** gene which normally converts NeuAc to NeuGc [24]. The concentrations of NeuGc-containing free oligosaccharides were highest in the colostrum samples but were reduced to trace amounts as lactation progressed (Figure 1). Throughout the lactation α2-3-linked oligosaccharides were in highest abundance among the sialylated oligosaccharides in both Holstein and Jersey cows (Figure 1). Levels of α2-6-linked sialylated oligosaccharides were relatively low in bovine milk. In contrast, α2-6-linked sialylated oligosaccharides are present in greater proportion than α2-3-linked sialylated oligosaccharides in human milk [25].

### Genes involved in milk oligosaccharide metabolism

An indication of the genes involved in bovine milk oligosaccharide metabolism was obtained by analyzing the oligosaccharide profiles of Holstein and Jersey cows at four different stages of lactation. The high abundance of sialylated and galactosylated oligosaccharides in bovine milk highlight the importance of genes coding for enzymes involved in metabolic processes related to sialylation and galactosylation of oligosaccharides in milk somatic cells. No fucosylated free oligosaccharides were identified in the present analysis or in the studies conducted by Barile *et al.* [26] and Tao *et al.* [14], [15]. However, one free fucosylated oligosaccharide was reported in Holstein colostrum by Saito *et al.* [27], and bovine milk protein analysis has shown the presence of N-linked and O-linked fucosylated oligosaccharides in mucins and other proteins [28]. Therefore, the genes coding for enzymes involved in metabolism of fucosylated oligosaccharides were also examined. The presence of GalNAc and GlcNAc as monomers in bovine milk oligosaccharides led to selection of genes involved in their metabolism for further characterization. Because all N-linked glycan synthesis is initiated by conjugation with mannose [29] genes involved in mannosylation were also examined.

**Figure 2:**
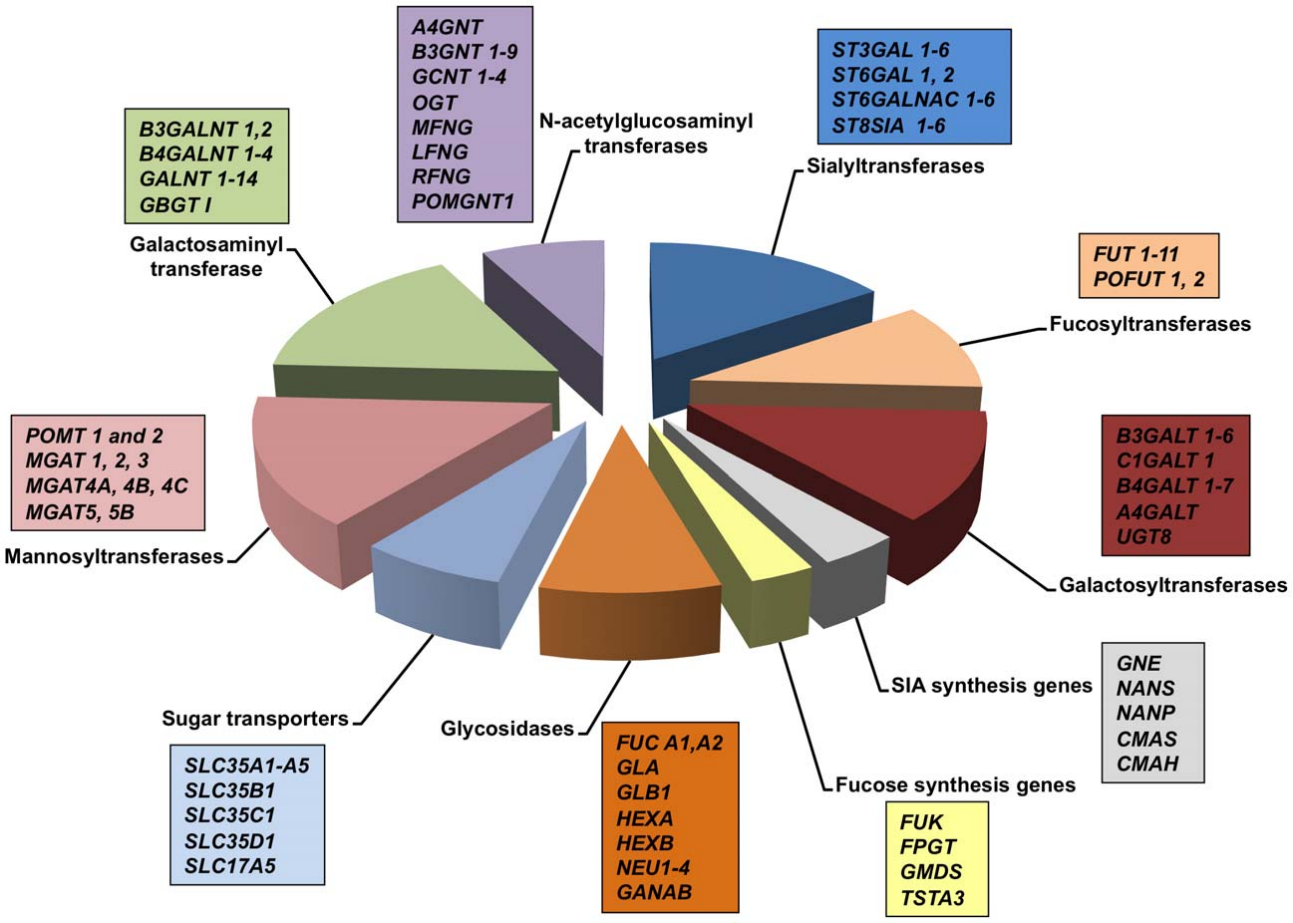
Diagram showing 128 genes from 10 functional oligosaccharide metabolic categories in mammals. The gene list was created by a detailed literature and databases (GlycoGene, Consortium for Functional Glycomics, KEGG GLYCAN, Carbohydrate-Active enzyme, Resources for Informatics of Glycomes at Soka, Glyco-Net) search. *ST6GALNAC3*, *ST6GALNAC4*, *FUT3*, *FUT6*, *FUT7*, *A4GALT* and *GALNT9* are not present in the Btau4.0 bovine genome assembly.

A list of genes that encode proteins involved in the metabolism of oligosaccharides containing sia, fucose, GalNAc, GlcNac GlcNAc and mannose was created by a detailed literature and databases search. The gene list consists of 128 genes belonging to ten functional oligosaccharide metabolic categories of sialyltransferases, fucosyltransferases, galactosyltransferases, sialic acid synthesis genes, fucose synthesis genes, glycosidasess, sugar transporters, mannosyltransferases, galatosaminyl transferases and N-acetylglucosaminyl transferases in mammals (Figure 2). When these genes are considered in bovines, *FUT5 (FUTB)* gene is readily identified as the orthologous homolog of the three human fucosyltrasferase genes, *FUT3*, *FUT5* and *FUT6* [30]. *ST6GALNAC3*, *ST6GALNAC4*, *A4GALT*, *GALNT4* and *FUT7* were not identified in the bovine assembly in NCBI Entrez gene database (http://www.ncbi.nlm.nih.gov/gene/) and Ensemble database (http://uswest.ensembl.org).

### RNA-Seq gene expression analysis and GO annotation

Expression profiling of genes in the milk oligosaccharide metabolism pathway was conducted by next generation RNA sequencing (RNA-Seq) using Illumina IG technology [31]. The advantage of RNA-Seq to measure gene expression over other methods, like microarrays, is that RNA-Seq is a probe independent platform and enabled expression profiling of all the glycosylation-related genes in bovine. In contrast the Affymetrix GeneChip® Bovine Genome Array contains probes for only 83 glycosylation-related genes. In addition, RNA-Seq provided a high dynamic range and additional genome structure information such as alternative splice forms and single nucleotide polymorphisms **(SNP)**.

The detailed chemical analyses of oligosaccharides identified the free oligosaccharides present in colostrum and characterized their sharp decrease through the progress of lactation. Unfortunately, high-quality RNA could not be extracted from somatic cells in colostrum samples due to enrichment of proteins and contamination of samples with blood. Therefore, gene expression analysis was conducted between day 15 which was the stage with the second highest concentration of free milk oligosaccharides, and day 250 milk samples.

By analysis of the unique gene reads and the unique exon reads, an RPKM value of 0.3 was set as the threshold for detectable gene expression above background. The *de novo* assembly and GO annotation identified novel exons in *B4GLAT7*, *MGAT4B*, *ST3GAL1*, *ST6GAL1* and *ST8SIA4* genes; however novel glycosylation-related genes were not identified. Therefore, the gene expression analysis was conducted in the 121 glycosylation-related genes present in the Btau4.0 bovine genome assembly (Figure 2).

### Overview of glycosylation-related gene expression analysis

Twenty-nine genes related to milk oligosaccharide metabolism had RPKM values below the threshold in all the milk samples (n = 12) and these genes were considered as not expressed in the bovine milk (Table S1). Ninety-two genes involved in milk oligosaccharide metabolism were expressed (Table S1) in at least one of the milk samples (n = 12). All sugar transporters, fucose synthesis genes and sialic acid synthesis genes shown in Figure 2, were expressed in bovine milk. However, not all of the glycosyltransferases were expressed (Table S1). This indicates a selective expression of glycosyltransferases. The glycosidases, *NEU2* and *NEU4* were not expressed in bovine milk. In humans, *NEU2* is expressed only in skeletal muscles and *NEU4* is ubiquitously expressed in various tissues [32], [33]. However, no studies have been conducted on the expression of these genes in human milk or mammary gland tissues.

The ninety-two genes expressed in the milk samples had RPKM values ranging from 0.3 to 172.2. Most of these genes showed low (0.3–10 RPKM) to moderate (10–50 RPKM) expression in bovine milk (Table S1). In both the Holstein and Jersey breeds, *B4GALT1* and *TSTA3* showed the highest expression in transition milk samples. *B4GALT1* encodes an enzyme that participates in glyconjugation and lactose biosynthesis which occurs exclusively in the mammary gland [34]. Since the milk yield is higher in the transition stage of lactation than in late lactation, there is increased lactose biosynthesis during earlier stages of lactation than at terminal stages. This is reflected in the increased expression of *B4GALT1* in transition milk samples. *TSATA3* encodes for an enzyme in the *de novo* pathway of fucose synthesis. This is the first time that *TSTA3* expression has been measured in a mammalian milk sample and the high expression of this gene indicates an increase in fucose synthesis in the milk cells during the initial stages of lactation. The bovine oligosaccharide profile analysis showed no abundant free fucosylated oligosaccharides in milk. However, glyoproteomic analyses of bovine milk have shown many fucosylated oligosaccharides conjugated with milk fat globular membrane proteins such as mucins [28], [35]. Therefore, the high expression of *TSTA3* is an indication of the presence of fucosylated conjugates in bovine milk.

The *HEXA* gene had the highest expression in late lactation milk samples from both the Holstein and Jersey breeds. The enzyme hexosaminidase encoded by the *HEXA* gene has shown high activity in human milk and is responsible for cleaving glucosamine and galactosamine from gangliosides and other glycoconjugates [36]. The high expression of the *HEXA* gene in late lactation of the Holstein and Jersey milk samples indicates an increased catabolism of glycoconjugates containing terminal glycosamine and galactosamine.

There was a remarkable similarity in the expression pattern of the ninety-two genes between the two breeds and none of the genes showed statistically significant changes in expression between Holstein and Jersey cows (p≤0.05 and FDR q≤0.3). These expression results agree with the free oligosaccharide profiles in milk where no significant changes of free oligosaccharides were observed between the two breeds.

In both Holstein and Jersey cows most of the genes in oligosaccharide metabolism had high expression during the late lactation. Only seven genes in Holstein cows and six genes in Jersey cows had more than two-fold higher RPKM expression values in transition milk (Figure S1). Sixty-three genes in Holstein cows and 48 genes in Jersey cows had more than two-fold higher RPKM expression values in late lactation milk (Figure S1). Statistically significant gene expression changes were observed in Holstein cows between transition and late lactation milk (p≤0.05 and FDR q≤0.3). Even though the expression patterns of the genes were similar to Holstein cows, statistical significant gene expression was not observed in Jersey cows between transition and late lactation milk (p≤0.05 and FDR q≤0.3).

### Expression of genes encoding enzymes in sialylated oligosaccharide metabolism

**Figure 3:**
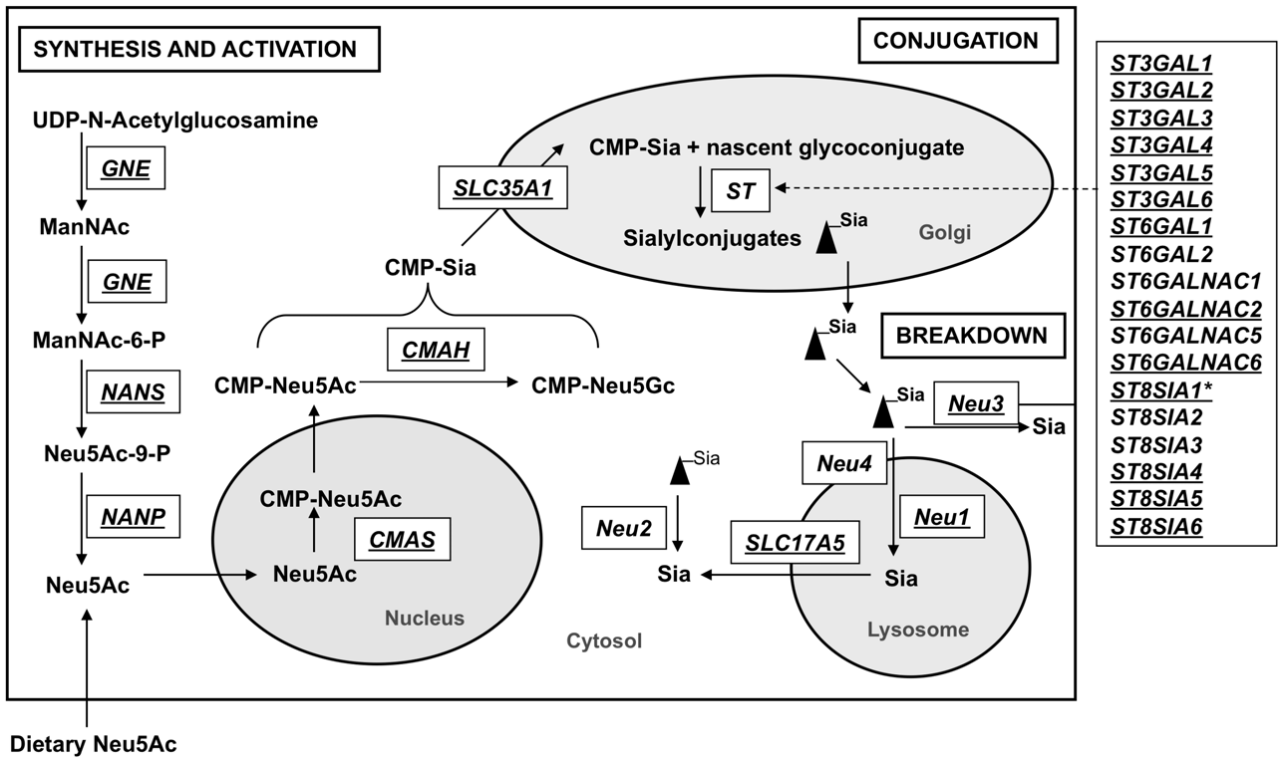
Sialic acid metabolism pathway. Important genes involved in sia synthesis, activation, conjugation and breakdown are depicted in the boxes. Sialic acid synthesis starts in the cytosole and then sia is activated in the nucleus. Activated sia is transported to Golgi for conjugation and modification. These sialylated conjugates are cleaved by neuraminidases found in the lysosomes, cytosole and the cell membrane. The genes expressed in Holstein/Jersey milk are underlined. Among the expressed genes only *ST8SIA1* (marked with an asterisk) had a higher expression in the transition milk. All other genes had higher expression in late lactation.

Twenty-nine genes from the list of 121 genes were identified as genes important for metabolism of sialylated oligosaccharides in milk. The metabolic pathways of sialylated oligosaccharides initiate with the biosynthesis of sialic acid in the cytosol by conversion of UDP-N-acetylglucosamine (UDP-GlcNAc) to N-acetylmannosamine (ManNAc) followed by phosphorylation to ManNAc-6-phosphate by the bifuncational enzyme UDP-N-acetylglucosamine-2-epimerase/N-acetylmannosamine kinase (*GNE*). ManNAc-6-P is converted to NeuAc-9-phosphate by sialic acid 9-phosphate synthase (*NANS.* The product is then dephosphorylated and converted into Neu5Ac by sialic acid 9-phosphate phosphatase *(NANP)*. Neu5Ac is then imported into the nucleus through a nuclear pore and converted into CMP-Neu5Ac by the enzyme CMP-Sia synthase (*CMAS*). Synthesized CMP-Neu5Ac is exported back into the cytosol via nuclear pores and then transported into the Golgi apparatus via the transporter protein SLC35A1 for further modification and conjugation by sialyltransferases *(ST)*. After their half-life, the removal of α-glycosidically linked sia residues from glycoconjugates is catalyzed by neuraminidases (*NEU1*-*NEU4*) (Figure 3) [37].

**Figure 4:**
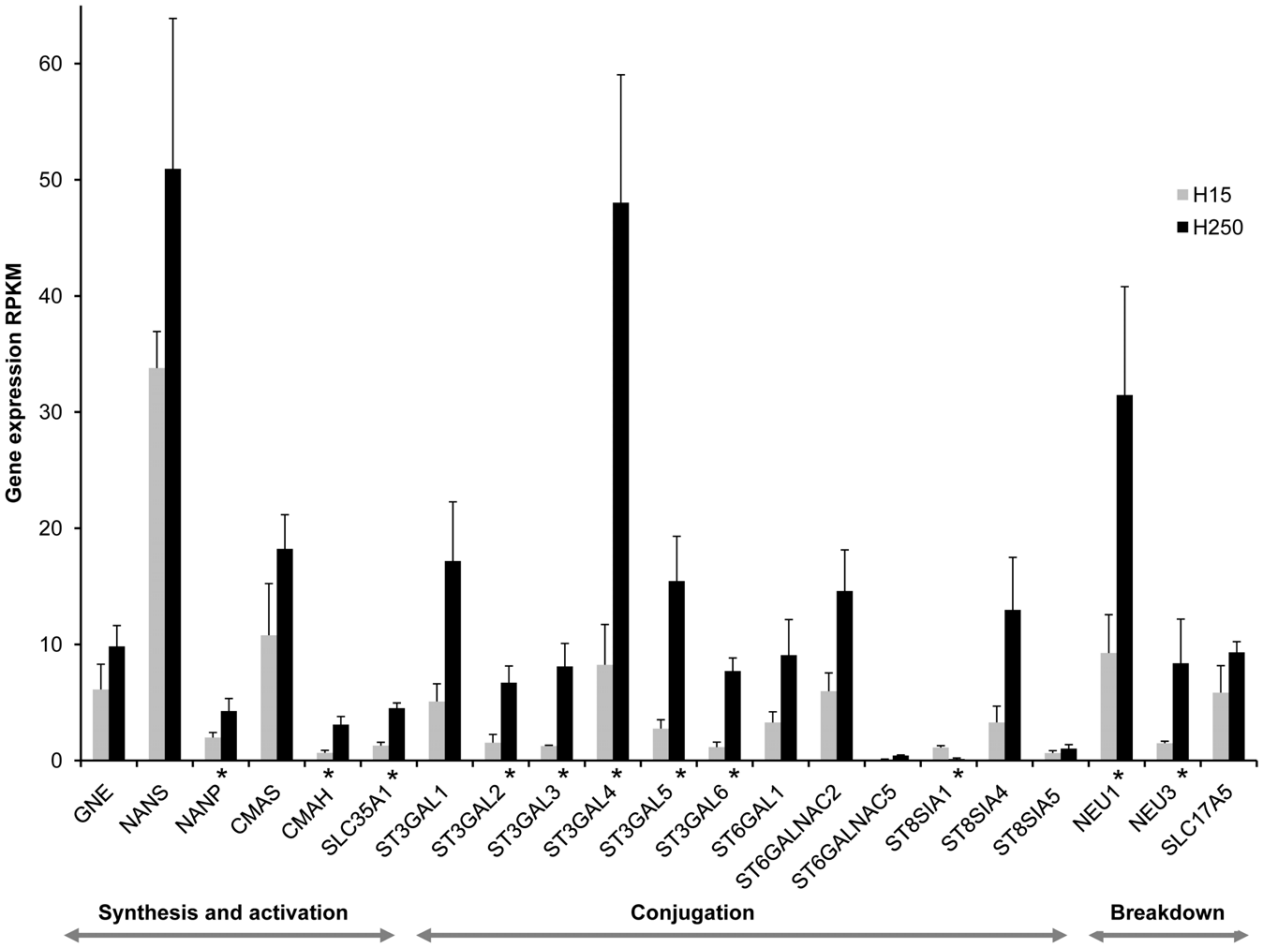
Expression profiles of the genes encoding enzymes in sialylated oligosaccharides metabolism in Holstein milk samples. Genes with significant change in expression are marked with an asterisk (p≤0.05 and FDR q≤0.2). Same pattern of expression was observed in the Jersey milk samples. However in Jersey animals there were no genes with statistical significant change in expression between the transition and late lactation milk (p≤0.05 and FDR q≤0.2). α2,3-sialyltransferases (ST3GAL1-6) showed higher average levels of expression than α2,6-sialyltransferases (ST6GAL) in transition and late lactation milk.

Twenty-one genes in the sialylated oligosaccharide metabolism pathway had low to medium levels of expression in Holstein and Jersey milk (Figures 3 and 4). Six *ST*s, *NEU2* and *NEU4* were not expressed in any of the milk samples. Twenty of the expressed genes showed increased levels of expression in late lactation (Figure 4). Only *ST8SIA1*, which regulates synthesis of GD3, the most abundant ganglioside in early lactation bovine and human milk [38], showed a higher expression in transition milk. *ST35GAL5*, which regulates synthesis of GM3, a prominent ganglioside in late lactation [38], had high expression in late lactation milk. Among the sialyltransferase genes, α2,3-sialyltransferases showed higher average levels of expression than α2,6-sialyltransferases in transition and late lactation milk. The bovine oligosaccharide profile analysis indicated 3′-sialylactose to be more abundant than the 6′-sialylactose in transition and late lactation milk, and this gene expression pattern of α2,3 and α2,6-sialyltransferases agrees with the oligosaccharide profiles. However, these results contrasts with human milk where 6′ sialylactose is more abundant [25]. The logical prediction that in human milk there will be high expression of α2,6-sialyltransferases appears to be true (unpublished data Medrano lab). The reversal of the relative abundance of the type of sialylated oligosaccharides and the selective expression of sialyltransferases in bovine milk may be explained by different biological functions of cow milk and human milk. The higher expression of neuraminidases and sia transporters in late lactation milk indicate an increase in synthesis and breakdown of sialylconjugates in late lactation. The overall summary of the expression profiles of genes in metabolism of sialylated oligosaccharides indicates increased metabolism of sialylated conjugates in late lactation when compared with the transition milk. These findings agree with published research on the composition of bovine milk sialylconjugates in relation to the stages of lactation ([39] where sialylated conjugates were low in transition milk and increased in late lactation.

### Expression of genes encoding enzymes in fucosylated oligosaccharide metabolism

**Figure 5:**
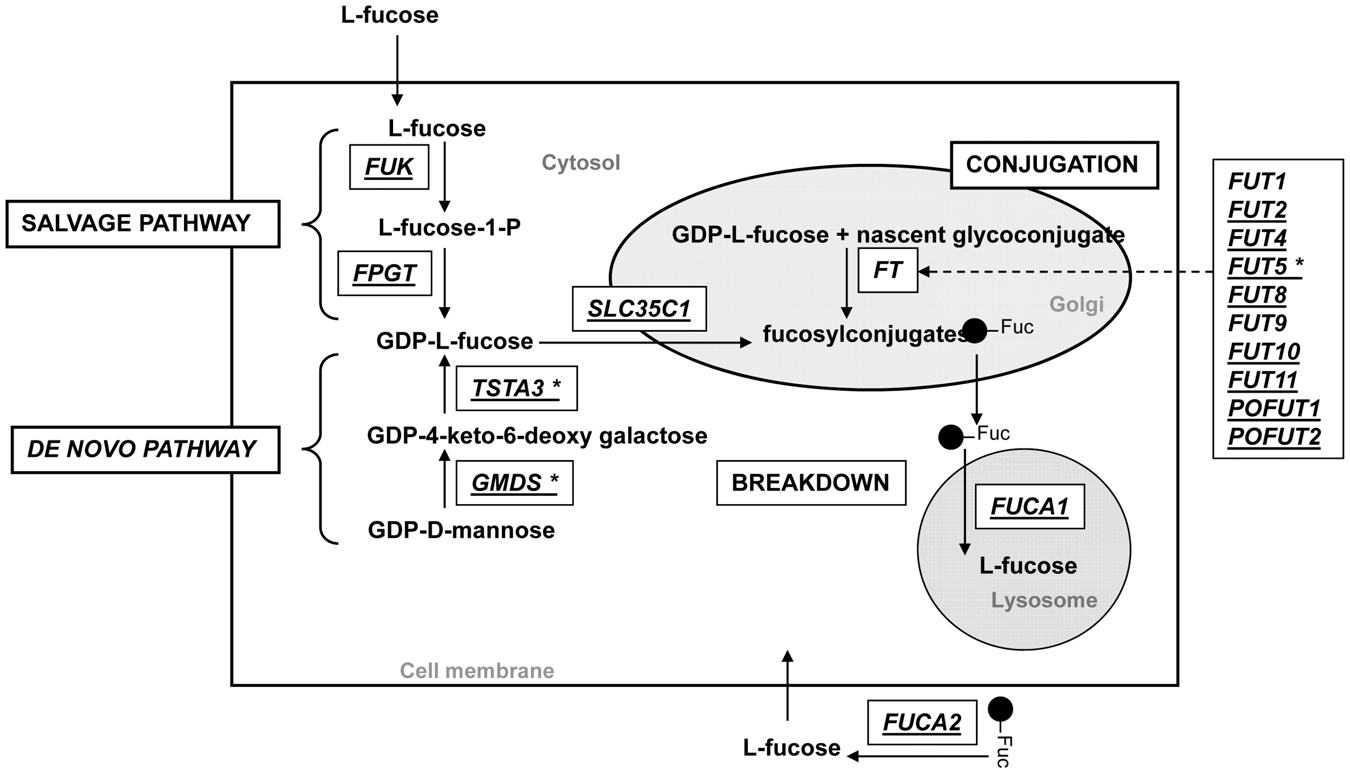
Fucose metabolism pathway. Genes encoding enzymes in fucose synthesis, conjugation and breakdown are depicted in the boxes. The two pathways of fucose synthesis occur in the cytosol. The synthesized GDP-L-fucose is transported to Golgi where they are conjugated with carbohydrates and modified in the Golgi apparatus. These fucosylated conjugates are cleaved by fucosidases. The genes expressed in Holstein milk are underlined. Among the expressed genes only *TSTA3*, *GMDS* and *FUT5* (marked with an asterisk) had a higher expression in the transition milk. The expression of *FUK* was similar in the two stages. All other genes had higher expression in late lactation.

Seventeen genes from the list of 121 genes were identified as genes important for metabolism of fucosylated oligosaccharides in bovine milk. The metabolic pathway of fucosylated oligosaccharides initiates with the biosynthesis of GDP-L-fucose in the cytoplasm. Two pathways are involved in the fucose biosynthesis process. The *de novo* pathway is constitutively expressed and converts GDP-D-mannose into GDP-L-fucose by the catalytic activities of the enzymes GDP-mannose-4, 6-dehydratase *(GMDS)* and GDP-L-fucose synthase *(TSTA3)*. In the salvage pathway, free fucose is obtained from degradation of fucosylated conjugates and converted to GDP-L fucose by the catalytic activities of the two enzymes L-fucokinase *(FUK)* and fucose pyrophosphorylase *(FPGT)*. GDP-L-fucose synthesized by these two pathways is transported into the Golgi apparatus via the transporter protein SLC35C1 for further modification and conjugation by fucosyaltransferases *(FUT)*. After their half-life, the removal of fucosylated conjugates are catalyzed by fucosidases, *FUCA1* and *FUCA2* (Figure 5) [40].

**Figure 6:**
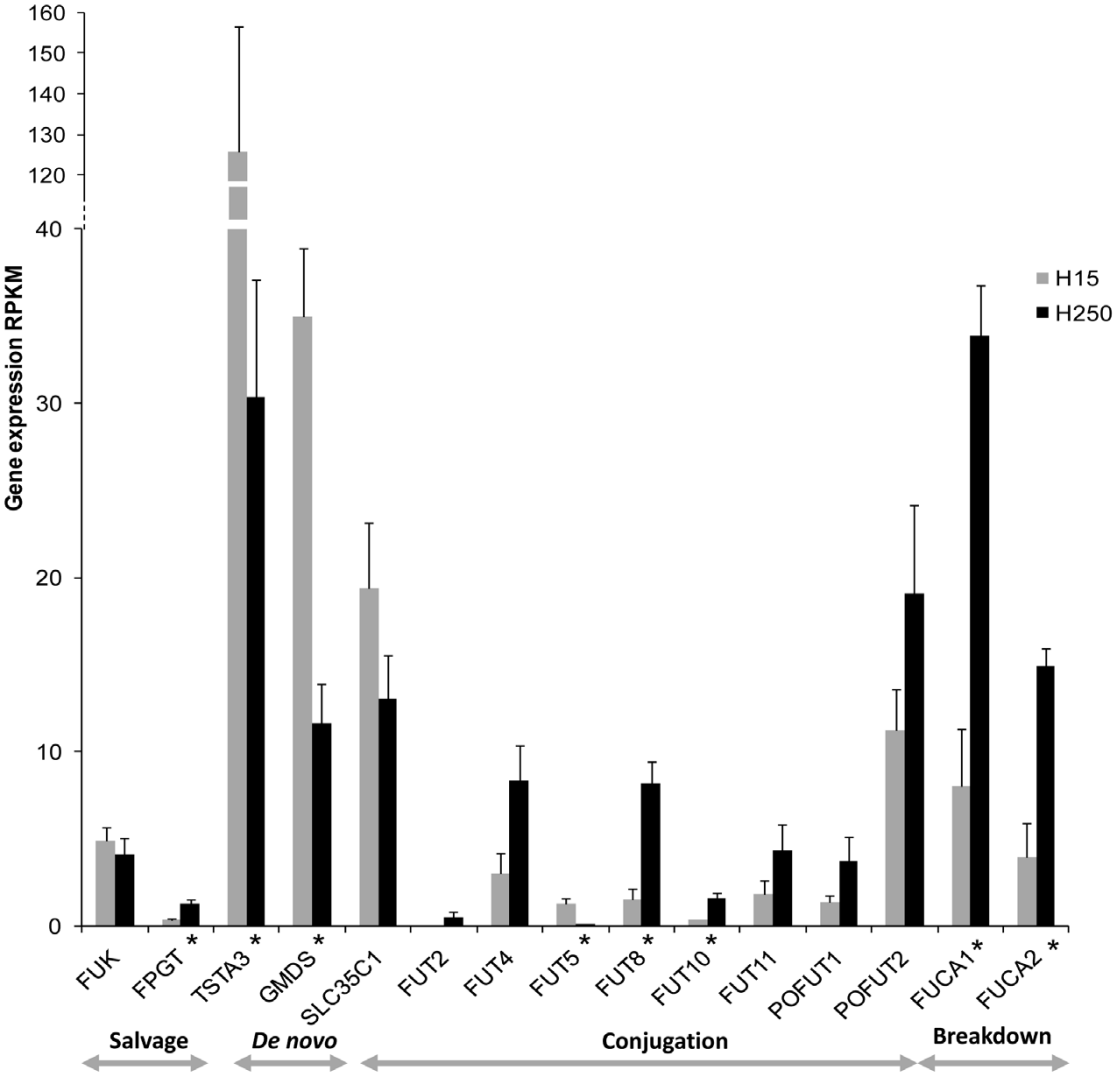
Expression profiles of the genes encoding enzymes in fucosylated oligosaccharides metabolism in Holstein milk samples. Genes with significant change in expression are marked with an asterisk (p≤0.05 and FDR q≤0.2). Except for *FUT5* same pattern of expression was observed in the Jersey milk samples. However in Jersey animals there were no genes with statistical significant change in expression between the transition and late lactation milk (p≤0.05 and FDR q≤0.2).

Fifteen genes in fucosylated oligosaccharide metabolism pathway (genes involved in synthesis, conjugation, transport and breakdown) were expressed in Holstein and Jersey milk samples (Figures 5 and 6). Except for *FUT5*, both breeds had the same pattern of expression between the two stages of lactation. *FUT5* was only expressed in the transition milk samples of Holstein animals. Expression values of *FUT5* were below the threshold level in late lactation Holstein samples and transition and late lactation Jersey samples. However, the gene expression of more Holstein and Jersey samples should be studied before drawing a definite conclusion on exclusive expression of *FUT5* in transition milk in Holstein cows.

The genes in the *de novo* synthesis pathway of fucose were highly expressed in transition milk samples. Studies conducted in mouse and human cell lines have shown a positive correlation of the expression of genes in *de novo* fucose synthesis pathway with the GDP-L-fucose levels in the cytosol [41]. Therefore, the increased expression of *TSTA3*, *GMDS* and *SLC35C1* indicates increased synthesis of GDP-L-Fucose in transition milk. Increased expression of genes in *de novo* synthesis of fucose and the low expression of fucosyltransferases in milk indicate an alternative biological pathway of fucose metabolism in milk or an unidentified isoform of a fucoyltransferase gene in bovine milk. Both *FUCA1* and *FUCA2* were expressed in bovine milk and similar to neuraminidases, the expressions of fucosidase genes were significantly high in the late lactation samples.

Even though the fucosylated free oligosaccharides were not identified in the present analysis, the overall gene expression analysis indicates the likely presence of fucosylated oligosaccharides in bovine milk. A possible explanation for not detecting fucosylated free oligosaccharides could be due to the degradation of large fucosylated free oligosaccharides by the fucosidases in milk. This is the first study that shows the expression of fucosidase genes and therefore the likely presence of fucosidase enzyme activity in the bovine milk. Fucosidases have shown to be fully active under the conditions in human milk and the activity of the enzymes shown an increase over the course of lactation [36]. More research is necessary to draw conclusions on the fucosidase activity, and its effect on degradation of fucosylated oligosaccharides in cow milk.

### Expression of galactosylated and N-linked oligosaccharide metabolism genes

The analysis of milk oligosaccharides showed a relative increase in the galactosylated oligosaccharides with the progress of lactation. Both Holstein and Jersey cows had increased expression of most of the galactosyltransferase genes in late lactation milk. Significant increases in the expression were observed in *B4GALT4*, *B4GALT5* and *B4GALT6* genes in late lactation milk. Among these genes, *B4GALT4* is responsible for synthesis of glycoproteins, especially mucin-type core 2 branching [34], *B4GALT5* is involved in the synthesis of N-linked oligosaccharides in many glycoproteins [42] and *B4GALT6* encodes for the enzyme lactosyl ceramide synthase that is required for apoptosis ([43]. Compared to β-1,4-galactosyltransferases the average expression was low for β-1,3-galactosyltransferases in both breeds. Late lactation milk had increased expression of two galactosidases, *GLA* and *GLB1*. Both these genes encode enzymes that cleave terminal galactose residues from gangliosides and other glycoconjugates ([44].

Most of the glucosaminyl transferases, galactosaminyl transferases and mannosyltransferases had higher expression in late lactation milk samples indicating an increase in the protein bound N-linked oligosaccharides during late lactation. All the expressed glycosidases in milk showed increased expression during late lactation indicating an enhanced catabolism of glycoconjugates during late lactation.

### Correlation between oligosaccharide profiles and glycosylation-related gene expression

No significant differences were observed between Holsteins and Jerseys in free oligosaccharides profiles and similarly, none of the glycosylation-related genes showed statistically significant differences in expression level between the two breeds. However, between different stages of lactation significant changes were observed in both, oligosaccharide profiles and gene expression levels.

The oligosaccharide profile analysis showed higher degree of sialylation of free oligosaccharides in transition milk, and only in late lactation did we observe higher expression of most of the genes in sialylated oligosaccharides metabolism. This high gene expression in late lactation suggests a likely increase in the synthesis and breakdown of bound sialylated conjugates at this stage. At all stages of lactation 3′-sialylactose was more abundant than the 6′-sialylactose in bovine milk, and as expected, α2,3-sialyltransferase genes had higher expression levels.

Even though fucosylated free oligosaccharides were not identified in the profile analysis, the gene expression analysis suggests the presence of fucosylated components in bovine milk. We detected expression of genes coding forfucosidase enzymes that can degrade the fucosylated free oligosaccharides in milk, which may explain the absence of fucosylated free oligosaccharides in the profile analysis. There was a relative increase in the galactosylated oligosaccharides with the progress of lactation. Similarly the gene expression profiles also showed increased expression of most of the galactosyltrasnsferase genes in late lactation milk.

### Conclusions

This is the first published study on expression profiling of genes involved in milk oligosaccharide metabolism in any mammalian species. The study compared the annotation of bovine milk oligosaccharide profiles with the expression analysis of genes in milk oligosaccharide metabolism. Results showed that the majority of the genes involved in oligosaccharide metabolism are expressed in the milk somatic cells. No significant differences in gene expression were observed between the Holstein and Jersey breeds. However, a significant change in gene expression was observed in relation to stage of the lactation where most of the genes were more highly expressed in late lactation milk samples. This study provides considerable insight into bovine milk glycobiology and its underlying genetic mechanisms. The candidate genes coding for enzymes involved in sialic acid and fucose metabolism and the mechanism of their regulation through lactation need to be further characterized. These results will allow the design of targeted breeding strategies to guide the content and composition of beneficial oligosaccharides in bovine milk.

## Supporting Information

Figure S1 **1a:** Genes with ≥2 fold increased expression in transition milk. Genes with statistically significant expression changes (p≤0.05 and FDR q≤0.3)) in the Holstein breed are marked with an asterisk (*). The Jersey cows did not have any genes with statistically significant changes in expression between transition and late lactation milk. **1b:** Genes with ≥2 fold increased expression in late lactation milk. Genes with statistically significant expression changes (p≤0.05 and FDR q≤0.3)) in the Holstein breed are marked with an asterisk (*). The Jersey cows did not have any genes with statistically significant changes in expression between transition and late lactation milk.
(TIF)

Table S1 RNA-Seq gene expression values (RPKM) in milk somatic cells for the 121 genes involved in oligosaccharide metabolism at two stages of lactation (days 15 and 250) in Holstein and Jersey cows.
(DOCX)
