# Supplementary figures and images for: Transcriptome Profiling of Bovine Milk Oligosaccharide Metabolism Genes Using RNA-Sequencing
Source: PLoS One. 2011 Apr 25;6(4):e18895. doi: 10.1371/journal.pone.0018895 (PMC3081824; doi:10.1371/journal.pone.0018895)

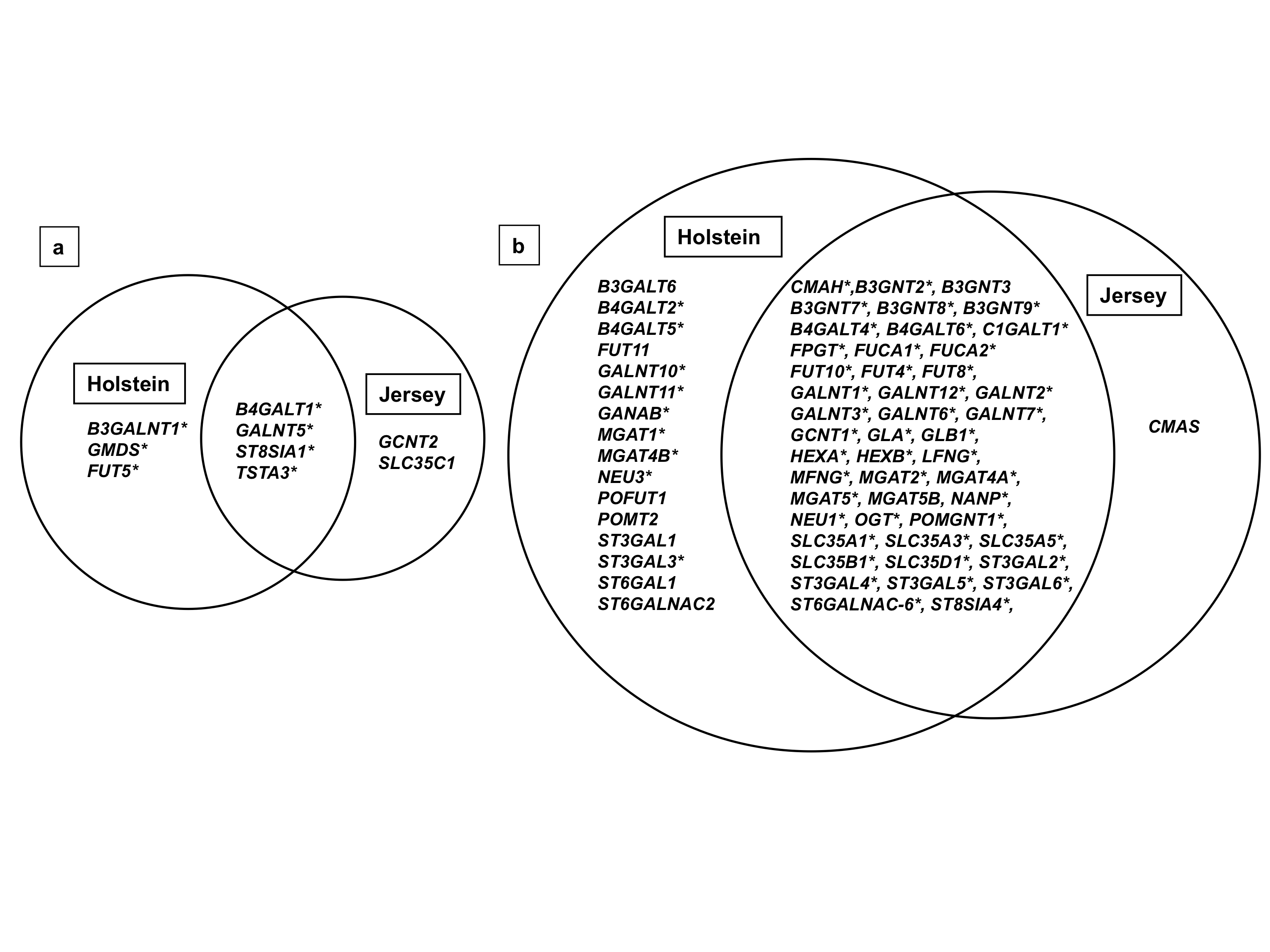

Supplement: Figure S1 — 1a: Genes with ≥2 fold increased expression in transition milk. Genes with statistically significant expression changes (p≤0.05 and FDR q≤0.3)) in the Holstein breed are marked with an asterisk (*). The Jersey cows did not have any genes with statistically significant changes in expression between transition and late lactation milk. 1b: Genes with ≥2 fold increased expression in late lactation milk. Genes with statistically significant expression changes (p≤0.05 and FDR q≤0.3)) in the Holstein breed are marked with an asterisk (*). The Jersey cows did not have any genes with statistically significant changes in expression between transition and late lactation milk. (TIF) [file pone.0018895.s001.tif]
