# Supplementary material for: Transcriptome Profiling of Bovine Milk Oligosaccharide Metabolism Genes Using RNA-Sequencing
Source: PLoS One. 2011 Apr 25;6(4):e18895. doi: 10.1371/journal.pone.0018895 (PMC3081824; doi:10.1371/journal.pone.0018895)
Supplement: Table S1 — RNA-Seq gene expression values (RPKM) in milk somatic cells for the 121 genes involved in oligosaccharide metabolism at two stages of lactation (days 15 and 250) in Holstein and Jersey cows. (DOCX) [file pone.0018895.s002.docx]

| **Gene symbol^1^** | **BTA^2^** | **H15^3^** | **H250^4^** | **J15^5^** | **J250^6^** |
| --- | --- | --- | --- | --- | --- |
| **Sialyltransferases** |  |  |  |  |  |
| ST3GAL1 | 14 | 5.1 | 17.2 | 5.0 | 9.9 |
| ST3GAL2 | 18 | 1.5 | 6.7 | 1.4 | 3.9 |
| ST3GAL3 | 3 | 1.3 | 8.1 | 4.4 | 4.7 |
| ST3GAL4 | 29 | 8.2 | 48.0 | 12.1 | 57.1 |
| ST3GAL5 | 11 | 2.8 | 15.5 | 2.5 | 12.8 |
| ST3GAL6 | 1 | 1.2 | 7.7 | 0.9 | 5.1 |
| ST6GAL1 | 1 | 3.3 | 9.1 | 4.0 | 4.6 |
| ST6GAL2 | 11 | 0.0 | 0.0 | 0.0 | 0.0 |
| ST6GALNAC1 | 19 | 0.0 | 0.0 | 0.0 | 0.0 |
| ST6GALNAC2 | 19 | 6.0 | 14.6 | 7.6 | 9.1 |
| ST6GALNAC5 | 3 | 0.0 | 0.0 | 0.1 | 0.1 |
| st6GalNAc6 | 22 | 0.1 | 0.4 | 0.0 | 0.1 |
| ST8SIA1 | 5 | 1.1 | 0.1 | 0.6 | 0.1 |
| ST8SIA2 | 21 | 0.0 | 0.0 | 0.0 | 0.0 |
| ST8SIA3 | 24 | 0.0 | 0.0 | 0.0 | 0.0 |
| ST8SIA4 | 7 | 3.3 | 13.0 | 2.5 | 8.4 |
| ST8SIA5 | 24 | 0.7 | 1.0 | 0.8 | 1.2 |
| ST8SIA6 | 13 | 0.0 | 0.0 | 0.0 | 0.0 |
| **SIA synthesis genes** |  |  |  |  |  |
| GNE | 8 | 6.1 | 9.8 | 4.5 | 6.4 |
| NANP | 13 | 2.0 | 4.3 | 1.9 | 5.4 |
| NANS | 8 | 33.8 | 50.9 | 43.6 | 62.0 |
| CMAS | 5 | 10.8 | 18.2 | 9.3 | 30.2 |
| CMAH | 23 | 0.7 | 3.1 | 0.6 | 3.3 |
| **Fucosyltransferases** |  |  |  |  |  |
| FUT1 | 18 | 0.0 | 0.1 | 0.0 | 0.1 |
| FUT2 | 18 | 0.0 | 0.5 | 0.0 | 0.8 |
| FUT4 | 15 | 3.0 | 8.3 | 3.3 | 7.9 |
| FUT5 | 7 | 1.3 | 0.0 | 0.3 | 0.3 |
| FUT8 | 10 | 1.6 | 8.2 | 0.8 | 5.8 |
| FUT9 | 9 | 0.0 | 0.0 | 0.0 | 0.0 |
| FUT10 | 27 | 0.3 | 1.6 | 0.3 | 1.0 |
| FUT11 | 28 | 1.9 | 4.3 | 1.8 | 3.0 |
| POFUT1 | 13 | 1.4 | 3.7 | 1.6 | 1.5 |
| POFUT2 | 1 | 11.2 | 19.1 | 17.5 | 16.7 |
| **Fucose synthesis genes** |  |  |  |  |  |
| FUK | 18 | 4.9 | 4.2 | 4.7 | 4.1 |
| FPGT | 3 | 0.4 | 1.3 | 0.3 | 0.7 |
| GMDS | 23 | 35.0 | 11.6 | 39.2 | 23.4 |
| TSTA3 | 14 | 121.4 | 30.4 | 172.2 | 51.0 |
| **Sugar transporters** |  |  |  |  |  |
| SLC35A1 | 9 | 1.3 | 4.5 | 1.2 | 5.9 |
| SLC35A2 | X | 7.1 | 10.8 | 10.0 | 7.1 |
| SLC35A3 | 3 | 1.1 | 2.9 | 0.3 | 1.7 |
| SLC35A4 | 7 | 15.2 | 22.1 | 14.1 | 11.4 |
| SLC35A5 | 1 | 1.0 | 5.4 | 1.0 | 4.5 |
| SLC35B1 | 19 | 26.3 | 73.6 | 26.7 | 64.4 |
| SLC35C1 | 15 | 19.4 | 13.1 | 17.0 | 6.5 |
| SLC35D1 | 8 | 0.3 | 1.2 | 0.2 | 1.0 |
| SLC17A5 | 9 | 5.9 | 9.3 | 3.8 | 6.6 |
| **Glycosidases** |  |  |  |  |  |
| FUCA1 | 2 | 8.0 | 33.9 | 8.0 | 29.0 |
| FUCA2 | 9 | 4.0 | 14.9 | 2.5 | 14.7 |
| GLA | X | 8.0 | 32.4 | 6.3 | 20.6 |
| GLB1 | 22 | 13.1 | 71.8 | 13.0 | 42.9 |
| HEXA | 10 | 27.6 | 106.9 | 16.8 | 101.9 |
| HEXB | 20 | 10.2 | 36.1 | 5.2 | 47.9 |
| NEU1 | 23 | 9.3 | 31.5 | 7.9 | 25.8 |
| NEU2 | Un.004.738 | 0.0 | 0.0 | 0.0 | 0.0 |
| NEU3 | 15 | 1.5 | 8.4 | 2.3 | 3.8 |
| NEU4 | 3 | 0.0 | 0.0 | 0.0 | 0.0 |
| GANAB | 29 | 29.2 | 76.0 | 29.5 | 34.2 |
| **Galactosyl transferases** |  |  |  |  |  |
| B3GALT1 | 2 | 0.0 | 0.0 | 0.0 | 0.0 |
| B3GALT2 | 16 | 0.0 | 0.0 | 0.0 | 0.0 |
| B3GALT3 | 10 | 0.0 | 0.0 |  | 0.0 |
| B3GALT4 | 23 | 2.4 | 2.4 | 3.7 | 2.8 |
| B3GALT5 | 1 | 0.1 | 0.3 | 0.0 | 0.2 |
| B3GALT6 | 16 | 1.3 | 2.7 | 2.1 | 2.3 |
| C1GALT1 | 4 | 2.2 | 8.0 | 1.6 | 6.6 |
| B4GALT1 | 8 | 140.0 | 43.5 | 164.2 | 21.9 |
| B4GALT2 | 3 | 7.0 | 19.8 | 9.6 | 15.0 |
| B4GALT3 | 3 | 5.9 | 9.1 | 6.8 | 6.9 |
| B4GALT4 | 1 | 0.7 | 6.9 | 1.2 | 4.7 |
| B4GALT5 | 13 | 4.5 | 17.3 | 6.9 | 13.4 |
| B4GALT6 | 24 | 1.9 | 14.7 | 1.3 | 7.1 |
| B4GALT7 | 7 | 4.6 | 8.3 | 4.2 | 7.8 |
| UGT8 | 6 | 0.0 | 0.1 | 0.0 | 0.0 |
| **Galactosaminyl transferases** |  |  |  |  |  |
| B3GALNT1 | 1 | 0.3 | 0.1 | 0.5 | 0.3 |
| B3GALNT2 | 28 | 2.0 | 3.4 | 2.4 | 3.8 |
| B4GALNT1 | 5 | 0.1 | 0.0 | 0.0 | 0.0 |
| B4GALNT2 | 19 | 0.0 | 0.0 | 0.0 | 0.0 |
| B4GALNT3 | 5 | 0.0 | 0.0 | 0.0 | 0.0 |
| B4GALNT4 | Un.004.787 | 0.0 | 0.0 | 0.0 | 0.0 |
| GALNT1 | 24 | 5.0 | 24.8 | 4.2 | 18.9 |
| GALNT10 | 7 | 1.9 | 9.3 | 3.2 | 4.9 |
| GALNT11 | 4 | 3.5 | 8.1 | 4.3 | 8.0 |
| GALNT12 | 8 | 0.4 | 1.9 | 0.4 | 1.7 |
| GALNT13 | 2 | 0.0 | 0.0 | 0.0 | 0.0 |
| GALNT14 | 11 | 0.0 | 0.0 | 0.0 | 0.0 |
| GALNT2 | 28 | 4.3 | 15.8 | 3.0 | 10.5 |
| GALNT3 | 2 | 1.7 | 7.9 | 1.5 | 3.8 |
| GALNT4 | 5 | 0.0 | 0.1 | 0.1 | 0.0 |
| GALNT5 | 2 | 0.9 | 0.1 | 1.8 | 0.0 |
| GALNT6 | 5 | 5.2 | 31.6 | 4.2 | 17.5 |
| GALNT7 | 8 | 1.0 | 5.5 | 1.0 | 4.6 |
| GALNT8 | 5 | 0.0 | 0.0 | 0.0 | 0.0 |
| GBGT1 | 11 | 6.8 | 11.0 | 6.2 | 4.2 |
| POMGNT1 | 3 | 4.0 | 14.0 | 4.5 | 10.8 |
| **N-acetylglucosaminyl transferases** | |  |  |  |  |
| A4GNT | 1 | 0.0 | 0.0 | 0.0 | 0.0 |
| B3GNT1 | 29 | 2.2 | 3.6 | 1.7 | 2.3 |
| B3GNT2 | 29 | 1.6 | 13.9 | 1.6 | 7.1 |
| B3GNT3 | 7 | 0.2 | 1.1 | 0.2 | 1.3 |
| B3GNT4 | 17 | 0.0 | 0.0 | 0.0 | 0.0 |
| B3GNT5 | 1 | 0.1 | 0.1 | 0.0 | 0.1 |
| B3GNT6 | 15 | 0.0 | 0.0 | 0.0 | 0.0 |
| B3GNT7 | 2 | 0.6 | 2.5 | 0.3 | 1.2 |
| B3GNT8 | 18 | 0.1 | 0.3 | 0.1 | 1.3 |
| B3GNT9 | 18 | 0.2 | 0.7 | 0.1 | 0.6 |
| GCNT1 | 8 | 0.3 | 3.6 | 0.3 | 1.9 |
| GCNT2 | 23 | 0.9 | 1.0 | 0.9 | 0.4 |
| GCNT3 | 10 | 0.0 | 0.1 | 0.0 | 0.1 |
| GCNT4 | 10 | 0.3 | 0.5 | 0.5 | 0.7 |
| MFNG | 5 | 10.4 | 81.3 | 13.7 | 30.1 |
| OGT | X | 4.7 | 21.6 | 3.7 | 18.2 |
| LFNG | 25 | 11.4 | 56.4 | 16.7 | 40.2 |
| RFNG | 19 | 18.6 | 36.8 | 17.8 | 33.9 |
| **Mannosyl transferases** |  |  |  |  |  |
| POMT1 | 11 | 4.0 | 4.7 | 3.7 | 3.0 |
| POMT2 | 10 | 4.5 | 10.0 | 4.1 | 5.2 |
| MGAT1 | 7 | 17.9 | 41.4 | 17.2 | 30.4 |
| MGAT2 | 10 | 3.5 | 11.4 | 2.7 | 5.9 |
| MGAT3 | 5 | 0.2 | 0.0 | 0.1 | 0.0 |
| MGAT4A | 11 | 1.5 | 5.1 | 1.3 | 4.4 |
| MGAT4B | 7 | 14.3 | 42.8 | 15.7 | 28.4 |
| MGAT4C | 5 | 0.0 | 0.0 | 0.0 | 0.0 |
| MGAT5B | 19 | 0.3 | 0.6 | 0.2 | 0.5 |
| MGAT5 | 2 | 3.3 | 17.0 | 3.7 | 8.2 |

Table was generated according to the functional metabolic categories shown in Figure 2.

Gene symbol^1^= gene symbol according to National Center for Biotechnology Information (NCBI) nomenclature

BTA^2^ = bovine chromosome number

H15^3^ = average gene expression (in RPKM units) in day 15 milk samples in Holstein cows (n=3)

H250^4^ = average gene expression (in RPKM units) in day 250 milk samples in Holstein cows (n=3)

J15^5^ = average gene expression (in RPKM units) in day 15 milk samples in Jersey cows (n=3)

J250^6^= average gene expression (in RPKM units) in day 250 milk samples in Jersey cows (n=3)
